# Evaluation of volatile compound profiles and sensory properties of dark and pale beers fermented by different strains of brewing yeast

**DOI:** 10.1038/s41598-023-33246-4

**Published:** 2023-04-25

**Authors:** Justyna Paszkot, Alan Gasiński, Joanna Kawa-Rygielska

**Affiliations:** Department of Fermentation and Cereals Technology, Faculty of Biotechnology and Food Science, Wrocław University of Environmental and Life Sciences, 51-630 Wrocław, Poland

**Keywords:** Metabolic engineering, Applied microbiology

## Abstract

To evaluate the differences in the volatile compound profile of dark and pale beers fermented by different strains of brewer's yeast, gas chromatography with flame ionization detection and gas chromatography mass spectrometry analysis of eight beers was carried out. The prevalent group of compounds in all the beers analysed were alcohols (56.41–72.17%), followed by esters (14.58–20.82%), aldehydes (8.35–20.52%), terpenes and terpenoids (1.22–6.57%) and ketones (0.42–1.00%). The dominant higher alcohols were 2-methylpropan-1-ol, 3-methylbutanol, phenethyl alcohol, among aldehydes furfural, decanal, nonanal, and among esters ethyl acetate, phenylethyl acetate and isoamyl acetate. Beers fermented by the top-fermenting yeast *Saccharomyces cerevisiae var. diastaticus* had the highest volatile content. The addition of dark malt in wort production process had no effect on the total content of volatiles, but for some beers it caused changes in the total content of esters, terpenes and terpenoids. Variations in the total volatile content between beers fermented by different yeast strains are mainly due to esters and alcohols identified. Sensory analysis of beers allowed us to identify the characteristics affected by the addition of dark speciality malts in the production of wort and yeast strains used in the fermentation process.

## Introduction

Taste and aroma are important factors that shape consumers' perceptions of beer^1–3^. The main brewing raw materials are water, malt, and hop. Malt is a source of cereal, malt, caramel or roasted flavor compounds. Hops, in turn, add fresh citrus, fruit, or herbal notes to beer. An equally important factor that affects beer quality is the strain of brewer's yeast used in the technological process^4,5^. During the fermentation, in addition to ethanol and carbon dioxide, yeast produces a number of by-products that make the aroma profile of beer more complex compared to wort^6^. Therefore, it can be considered as a natural method of flavor and aroma enhancement—bioflavoring, which involves the use of microorganisms to improve sensory quality^7^.

Brewing raw materials and the conditions of the technological process influence the chemical composition of beer, which shapes its taste, aroma, and colour^3,6,8,9^. Sensory characteristics depends on raw materials and the fermentation process^1–3^. During the thermal processing of malt, mash and wort, Maillard reactions, caramelisation, and pyrolysis occur^8^. Non-enzymatic browning reactions involve a number of transformations between reducing sugars and amino acids. Both groups of compounds are key in the metabolic processes of yeast, so a reduction in their availability is a reason for changes in the fermentation course. Compounds generated during heat treatment also have a direct effect on the metabolism of microorganisms by inhibiting the activity of enzymes involved in the alcoholic fermentation pathway. Fermentation of dark worts rich in Maillard reaction and carmelization products results in beers with a different volatiles profile from pale beers^2,8^. Specialty malts used in the production of dark beer styles introduce compounds with inhibitory effects on yeast metabolism (furfural, 5-hydroxymethylfurfural, and melanoidins) into beer^5,10,11^. Furfural and 5-hydroxymethylfurfural have been shown to inhibit glycolytic enzyme activity and induce cellular DNA damage^12,13^. Furthermore, they also inhibit the activity of pyruvate dehydrogenase and aldehyde dehydrogenase, which are necessary for the synthesis of higher alcohols from amino acids (Ehrlich pathway)^5^. The final products of the Maillard reaction are macromolecular melanoidins. They exhibit chelating properties of magnesium ions, which play a key role in yeast metabolism due to their participation as a cofactors of enzymatic reactions. Moreover, they counteract the effects of cellular stress, and participate in gene expression, growth and proliferation of yeast cells^5,10,13^. Such effects on metabolic processes may be the reason for changes in the content of volatile fermentation by-products. Although Maillard reaction compounds were shown to affect the synthesis of key flavor compounds by yeast, there is limited information in the literature on the effect of using dark speciality malts on fermentation by different strains of brewing yeast, including uncoventional—*Saccharomyces cerevisiae* kveik type and *Saccharomyces cerevisiae var. diastaticus*.

The purpose of this study was to analyze the effect of dark specialty malts and brewer's yeast strain on the volatile compound profile and sensory characteristics of beers. The novelty of the research was the comparison of the volatile compound profile and sensory evaluation results of beers fermented by classic top-fermenting and bottom-fermenting brewing yeast strains, as well as unconventional yeast strains in the ethanol fermentation of pale and dark wort made with the addition of dark speciality malt. The results of this research have enabled the expansion of current knowledge in the design of sensory properties of fermented beverages through the selection of biological material, which can contribute to increasing the sensory attractiveness of food products. Additionally, our research has identified the potential for the use of *Saccharomyces cerevisiae* kveik type yeast and *Saccharomyces cerevisiae var. diastaticus* in the production of dark beers, which has not been previously studied. The experimental material consisted of dark and pale beers fermented by bottom-fermenting yeasts *Saccharomyces pastorianus* and top-fermenting *Saccharomyces cerevisiae*, including unconventional kveik type brewing yeast and *Saccharomyces cerevisiae ver. diastaticus* yeast. The volatile compound profile was analysed by gas chromatography with flame ionising detection (GC-FID) and gas chromatography mass spectrometry (GC–MS). Sensory attributes of the beers were evaluated using a proprietary questionnaire by panelists trained in sensory analysis.

## Results and discussion

In our research, we subjected pale and dark beers made with different strains of top-fermenting (ale) and bottom-fermenting (lager) brewing yeast to comprehensive chromatographic analysis. Table 1 shows the total content of higher alcohols, esters, aldehydes, terpenes and terpenoids, ketones, and other compounds in beers. The GC-FID (Table 2) and GC–MS (Tables 3 and 4) methods were used to evaluate the content of 58 volatile compounds: 16 higher alcohols, 15 esters, 9 aldehydes, 4 ketones, 1 fatty acid, 1 epoxide, 1 alkane, and 1 diene. The proportion of chemical groups in the profile of volatile compounds of beers was shown in Table 1. The dominant group of compounds in all the beers analysed were alcohols (56.41–72.17%), followed by esters (14.58–20.82%), aldehydes (8.35–20.52%), terpenes and terpenoids (1.22–6.57%) and ketones (0.42–1.00%).

**Table 1:** Total contents and percentage share of groups of volatile compounds in beers.

Volatile compounds		Higher alcohols	Esters	Aldehydes	Terpenes and terpenoids	Ketones	Other compounds	Total volatiles
S04P	mg/L	76.27 ± 6.21 ^1^ b	17.05 ± 0.37 d	8.83 ± 0.05 b	2.65 ± 0.18 bc	0.44 ± 0.07 a	0.45 ± 0.08 b	105.69 ± 6.60 bc
	% TV	72.17	16.13	8.35	2.51	0.42	0.43	-
S04D	mg/L	76.61 ± 12.67 b	19.81 ± 0.98 c	13.30 ± 2.37 ab	2.01 ± 0.11 de	0.84 ± 0.43 a	1.32 ± 0.58 ab	114.00 ± 10.07 b
	% TV	67.20	17.38	11.66	1.86	0.74	1.16	-
S23P	mg/L	45.83 ± 2.35 cd	10.57 ± 0.04 e	10.22 ± 2.29 b	4.77 ± 0.51 a	0.53 ± 0.02 a	0.59 ± 0.07 b	72.51 ± 0.07 d
	% TV	63.21	14.58	14.10	6.57	0.73	0.81	-
S23D	mg/L	39.07 ± 2.89 d	11.31 ± 0.24 e	14.21 ± 4.26 ab	2.40 ± 0.49 cd	0.69 ± 0.01 a	1.46 ± 0.26 ab	69.25 ± 1.88 d
	% TV	56.41	16.33	20.52	3.64	1.00	2.11	-
KVP	mg/L	58.89 ± 4.30 c	18.57 ± 0.05 c	11.62 ± 1.14 ab	2.96 ± 0.22 b	0.84 ± 0.03 a	0.88 ± 0.08 ab	93.81 ± five.45 c
	% TV	62.77	19.80	12.39	3.20	0.90	0.94	-
KVD	mg/L	58.26 ± 4.12 c	18.82 ± 0.50 c	13.82 ± 1.59 ab	2.38 ± 0.22 cd	0.62 ± 0.30 a	1.78 ± 1.54 ab	95.75 ± 8.27 c
	% TV	60.84	19.66	14.43	2.56	0.65	1.86	-
SAP	mg/L	95.81 ± 1.12 a	22.48 ± 0.60 b	11.44 ± 1.34 ab	2.13 ± 0.07 cde	0.91 ± 0.00 a	1.53 ± 0.34 ab	134.33 ± 0.01 a
	% TV	71.33	16.74	8.52	1.60	0.68	1.14	-
SAD	mg/L	91.87 ± 4.65 a	29.79 ± 0.93 a	16.02 ± 1.90 a	1.69 ± 0.14 e	0.87 ± 0.16 a	2.79 ± 1.70 a	143.08 ± 4.00 a
	% TV	64.21	20.82	11.19	1.22	0.61	1.95	-

^1^Values are expressed as mean (n = 2) ± standard deviation. The mean values with different letters (a, b, c, d, e, f) within the same column are statistically different (*P* < 0.05). S04P—pale beer fermented by *Saccharomyces cerevisiae* S04 yeast strain, SO4D—dark beer fermented by *Saccharomyces cerevisiae* S04 yeast strain, S23P -pale beer fermented by *Saccharomyces pastorianus* S23 yeast strain, S23D—dark beer fermented by *Saccharomyces pastorianus* S23 yeast strain, KVP—pale beer fermented by *Saccharomyces cerevisiae* kveik type yeast strain, KVD—dark beer fermented by *Saccharomyces cerevisiae* kveik type yeast strain, SAP—pale beer fermented by *Saccharomyces cerevisiae var. diastaticus* yeast strain, SAD—dark beer fermented by *Saccharomyces cerevisiae var. diastaticus* yeast strain.

**Table 2:** Concentration of volatile compounds identified in beers by GC-FID.

Compound	Chemical group	S04P	S04D	S23P	S23D	KVP	KVD	SAP	SAD
		mg / L							
1-propanol	Alcohols	11.12 ± 1.51 ab^1^	9.45 ± 1.31 bc	7.51 ± 0.02 de	5.75 ± 0.48 e	8.46 ± 0.04 cd	8.55 ± 0.72 cd	12.21 ± 0.29 a	10.73 ± 0.01 ab
2-metylo-propan-1-ol	Alcohols	21.84 ± 2.71 a	21.58 ± 1.86 a	9.36 ± 0.15 c	6.37 ± 0.43 d	11.56 ± 0.14 c	11.07 ± 0.74 c	14.65 ± 0.35 b	11.84 ± 0.14 c
2-methylbutanol	Alcohols	6.61 ± 0.75 c	6.63 ± 0.42 c	6.51 ± 0.08 c	4.99 ± 0.27 d	5.53 ± 0.01 d	5.39 ± 0.34 d	9.68 ± 0.30 a	8.81 ± 0.19 b
3-methylbutanol	Alcohols	17.49 ± 2.02 bc	16.55 ± 1.54 cd	14.55 ± 0.02 d	11.46 ± 0.92 e	18.39 ± 0.08 bc	19.54 ± 1.16 b	39.74 ± 0.64 a	37.52 ± 0.43 a
Phenylethyl alcohol	Alcohols	16.73 ± 0.89 a	18.80 ± 7.39 a	4.93 ± 1.93 b	5.62 ± 1.47 b	12.05 ± 3.92 ab	11.01 ± 0.71 ab	16.50 ± 0.28 a	20.15 ± 5.57 a
Acetaldehyde diethyl acetal	Aldehydes	0.95 ± 0.10 d^1^	0.64 ± 0.01 e	1.11 ± 0.01 c	0.60 ± 0.03 e	1.03 ± 0.05 cd	2.12 ± 0.05 b	1.04 ± 0.02 cd	2.23 ± 0.02 a
Furfural	Aldehydes	3.01 ± 0.34 d	2.60 ± 0.25 d	2.88 ± 0.53 d	3.79 ± 0.31 cd	6.32 ± 0.73 ab	6.88 ± 0.61 a	5.13 ± 0.01 bc	7.77 ± 1.40 a
Ethyl acetate	Esters	9.98 ± 0.19 d	10.2 ± 0.24 d	6.39 ± 0.01 e	6.01 ± 0.70e	13.83 ± 0.08 c	13.78 ± 0.29 c	16.54 ± 0.58 b	20.07 ± 0.93 a
Isoamyl acetate	Esters	0.64 ± 0.03 d	0.70 ± 0.01 c	0.54 ± 0.00 e	0.54 ± 0.02 e	0.57 ± 0.00 e	0.61 ± 0.00 de	0.88 ± 0.03 b	1.05 ± 0.04 a
Ethyl hexanoate	Esters	0.06 ± 0.01 d	0.08 ± 0.01 d	0.16 ± 0.00 c	0.10 ± 0.03 d	0.10 ± 0.00 d	0.11 ± 0.00 cd	0.30 ± 0.04 b	0.52 ± 0.04 a
Ethyl octanoate	Esters	0.62 ± 0.03 d	0.75 ± 0.02 cd	0.73 ± 0.01 cd	0.84 ± 0.11 bc	0.66 ± 0.00 d	0.75 ± 0.00 cd	0.98 ± 0.15 b	1.44 ± 0.01 a
Ethyl decanoate	Esters	nd^2^	nd	nd	nd	nd	nd	nd	1.60 ± 0.25 a

^1^Values are expressed as mean (n = 2) ± standard deviation. The mean values with different letters (a, b, c, d, e, f) within the same row are statistically different (*P* < 0.05). ^2^ nd -not detected.

**Table 3:** Concentration of alcohols, aldehydes and esters identified in beers by GC–MS.

Compound	Chemical group	S04P	S04D	S23P	S23D	KVP	KVD	SAP	SAD
		mg/L							
1-Hexanol	Alcohols	0.05 ± 0.00 c^1^	0.13 ± 0.17 ab	0.07 ± 0.02 bc	0.10 ± 0.03 a	0.06 ± 0.02 bc	0.12 ± 0.03 a	0.06 ± 0.02 bc	0.10 ± 0.00 ab
1-Heptanol	Alcohols	0.10 ± 0.01 a	0.12 ± 0.00 ab	0.13 ± 0.00 ab	0.12 ± 0.04 ab	0.08 ± 0.00 b	0.14 ± 0.03 ab	0.15 ± 0.02 ab	0.17 ± 0.06 a
1-Octen-3-ol	Alcohols	0.05 ± 0.00 ab	0.09 ± 0.01 a	0.05 ± 0.01 ab	0.09 ± 0.07 a	nd^2^	nd	nd	nd
2-Propyl-1-pentanol	Alcohols	0.02 ± 0.02 a	0.03 ± 0.01 a	0.04 ± 0.06 a	0.01 ± 0.01 a	nd	0.03 ± 0.01 a	nd	nd
1-Octanol	Alcohols	0.66 ± 0.07 ab	0.89 ± 0.12 a	0.46 ± 0.07 bc	0.67 ± 0.20 ab	0.49 ± 0.02 bc	0.50 ± 0.23 bc	0.34 ± 0.06 c	0.42 ± 0.05 bc
2-Nonanol	Alcohols	0.29 ± 0.06 c	0.27 ± 0.02 c	0.55 ± 0.06 a	0.44 ± 0.10 ab	0.24 ± 0.00 c	0.46 ± 0.05 ab	0.37 ± 0.04 bc	0.30 ± 0.03 c
1-Nonanol	Alcohols	nd	0.33 ± 0.03 a	0.27 ± 0.06 a	0.15 ± 0.07 b	nd	nd	nd	nd
1-Decanol	Alcohols	0.24 ± 0.06 c	0.31 ± 0.05 bc	0.25 ± 0.06 c	0.55 ± 0.10 a	0.24 ± 0.03 c	0.21 ± 0.05 c	0.14 ± 0.03 c	0.45 ± 0.12 ab
2-Undecanol	Alcohols	nd	0.09 ± 0.00 d	0.31 ± 0.01 a	0.23 ± 0.04 b	0.13 ± 0.01 cd	0.20 ± 0.01 bc	0.11 ± 0.00 cd	0.20 ± 0.09 bc
1-Dodecanol	Alcohols	1.07 ± 0.10 cde	1.21 ± 0.41 bcd	0.66 ± 0.18 e	2.26 ± 0.26 a	1.41 ± 0.00 bc	8.40 ± 0.02 de	1.67 ± 0.17 b	0.94 ± 0.04 cde
1-Tetradecanol	Alcohols	nd	0.14 ± 0.01 b	0.18 ± 0.06 ab	0.24 ± 0.04 a	0.24 ± 0.01 a	0.20 ± 0.02 ab	0.21 ± 0.03 a	0.25 ± 0.01 a
Octanal	Aldehydes	0.32 ± 0.03 a	0.58 ± 0.35 a	0.35 ± 0.09 a	0.49 ± 0.23 a	0.31 ± 0.05 a	0.34 ± 0.13 a	0.35 ± 0.06 a	0.34 ± 0.00a
Nonanal	Aldehydes	1.72 ± 0.15 a	2.79 ± 0.88 a	1.96 ± 0.66	2.69 ± 1.48 a	1.42 ± 0.12 a	1.65 ± 0.27 a	1.66 ± 0.41 a	1.74 ± 0.01 a
Tetradecanal	Aldehydes	0.08 ± 0.02 a	0.07 ± 0.05 a	0.11 ± 0.04 a	0.12 ± 0.09 a	0.14 ± 0.04 a	0.06 ± 0.01 a	0.10 ± 0.04 a	0.14 ± 0.00 a
Decanal	Aldehydes	2.11 ± 0.40 b	5.61 ± 1.26 a	3.11 ± 1.98 ab	5.43 ± 2.52 a	1.79 ± 0.08 b	2.14 ± 0.44 b	2.46 ± 0.85 ab	2.94 ± 0.60 ab
Undecanal	Aldehydes	0.28 ± 0.07 b	0.56 ± 0.16 a	0.34 ± 0.11 ab	0.51 ± 0.19 ab	0.24 ± 0.07 b	0.32 ± 0.08 ab	0.36 ± 0.06 ab	0.42 ± 0.06 ab
Dodecanal	Aldehydes	0.27 ± 0.05 b	0.35 ± 0.03 ab	0.27 ± 0.03 b	0.45 ± 0.17 a	0.28 ± 0.07 ab	0.24 ± 0.02 b	0.26 ± 0.05 b	0.33 ± 0.01 ab
Tridecanal	Aldehydes	0.08 ± 0.04 a	0.08 ± 0.01 a	0.08 ± 0.03 a	0.14 ± 0.02 a	0.10 ± 0.04 a	0.08 ± 0.01 a	0.08 ± 0.02 a	0.11 ± 0.00 a
Methyl 4-methylidenehexanoate	Esters	0.10 ± 0.01 b	0.10 ± 0.02 b	0.14 ± 0.00 a	0.15 ± 0.03 a	0.06 ± 0.02 b	0.07 ± 0.00 b	0.10 ± 0.00 b	0.06 ± 0.02 b
Ethyl heptanoate	Esters	0.08 ± 0.03 b	0.10 ± 0.01 b	0.12 ± 0.00 ab	0.10 ± 0.02 b	0.07 ± 0.02 b	0.12 ± 0.03 ab	0.16 ± 0.02 a	0.16 ± 0.02 a
Ethyl phenylacetate	Esters	nd	nd	nd	nd	0.06 ± 0.04 a	0.06 ± 0.01 a	nd	0.05 ± 0.00 a
Phenethyl acetate	Esters	5.00 ± 0.04 b	7.03 ± 0.71 a	1.81 ± 0.15 d	2.34 ± 0.56 cd	2.63 ± 0.13 cd	2.57 ± 0.18 cd	2.47 ± 0.11 cd	2.91 ± 0.21 c
Ethyl 9-decenoate	Esters	0.15 ± 0.01 fg	0.25 ± 0.04 e	0.17 ± 0.00 f.	0.65 ± 0.04 b	0.09 ± 0.01 g	0.32 ± 0.01 d	0.50 ± 0.05 c	0.97 ± 0.02 a
Ethyl dodecanoate	Esters	0.08 ± 0.02 b	0.17 ± 0.00 b	0.12 ± 0.03 b	0.15 ± 0.02 b	0.11 ± 0.01 b	0.10 ± 0.00 b	0.12 ± 0.02 b	0.54 ± 0.11 a
Isopropyl dodecanoate	Esters	0.17 ± 0.00 ab	0.17 ± 0.00 ab	0.18 ± 0.00 ab	0.16 ± 0.03 ab	0.19 ± 0.01 ab	0.14 ± 0.01 b	0.21 ± 0.07 a	0.14 ± 0.00 ab
Ethyl tetradecanoate	Esters	0.08 ± 0.01 d	0.11 ± 0.01 cd	0.15 ± 0.07 bc	0.20 ± 0.03 ab	0.14 ± 0.02 bcd	0.14 ± 0.00 bcd	0.15 ± 0.00 bcd	0.22 ± 0.00 a
Hexyl acetate	Esters	0.06 ± 0.02 ab	0.08 ± 0.03 a	0.01 ± 0.00 c	0.05 ± 0.00 abc	0.03 ± 0.00 bc	0.04 ± 0.00 bc	0.04 ± 0.00 bc	0.03 ± 0.00 bc
2-Methylbutyl isobutyrate	Esters	0.03 ± 0.05 ab	nd	0.05 ± 0.00 a	0.03 ± 0.01 ab	0.04 ± 0.00 ab	0.03 ± 0.01 ab	0.03 ± 0.01 ab	0.02 ± 0.01 ab

^1^ Values are expressed as mean (n = 2) ± standard deviation. The mean values with different letters (a, b, c, d, e, f) within the same row are statistically different (*P* < 0.05).

^2^ not detected.

**Table 4:** Concentration of ketones, terpenes and terpenoids and other compounds identified in beers by GC-MS.

Compound	Chemical group	S04P	S04D	S23P	S23D	KVP	KVD	SAP	SAD
		mg/L							
3-Octanone	Ketones	0.10 ± 0.01 a^1^	nd^2^	nd	nd	nd	nd	nd	0.09 ± 13.07 b
6-Methyl-5-hepten-2-one	Ketones	0.16 ± 0.04 a	0.23 ± 0.06 a	0.19 ± 0.00 a	0.24 ± 0.05 a	0.16 ± 0.02 a	0.28 ± 0.17 a	0.16 ± 0.03 a	0.11 ± 0.01 a
2-Nonanone	Ketones	0.06 ± 0.01 a	0.09 ± 0.04 a	0.11 ± 0.04 a	0.10 ± 0.01 a	0.06 ± 0.02 a	0.07 ± 0.05 a	0.06 ± 0.00 a	0.04 ± 0.05 a
Nerylacetone	Ketones	0.11 ± 0.01 c	0.52 ± 0.34 ab	0.23 ± 0.02 bc	0.35 ± 0.07 abc	0.62 ± 0.07 a	0.28 ± 0.07 bc	0.69 ± 0.03 a	0.64 ± 0.13 a
Caryophyllene	Sesquiterpenes	nd	nd	0.16 ± 0.01 a	nd	nd	nd	nd	nd
(E)-β-Farnesene	Sesquiterpenes	nd	nd	0.24 ± 0.01 a	nd	0.03 ± 0.02 b	nd	nd	nd
Humulene	Sesquiterpenes	nd	nd	0.48 ± 0.06 a	nd	nd	nd	nd	nd
Nerolidol	Sesquiterpenes	0.11 ± 0.01 ab	0.04 ± 0.01 ef	0.06 ± 0.01 de	0.08 ± 0.01 cd	0.09 ± 0.00 bc	0.12 ± 0.01 a	0.04 ± 0.01 ef	0.03 ± 0.01 f.
D-Limonene	Terpenes	0.20 ± 0.22 a	0.02 ± 0.03 a	0.10 ± 0.02 a	0.01 ± 0.01 a	nd	nd	nd	nd
Linalool	Terpenoids	1.63 ± 0.10 bc	1.53 ± 0.12 c	2.05 ± 0.12 a	1.70 ± 0.33 abc	1.94 ± 0.16 ab	1.59 ± 0.13 bc	1.56 ± 0.08 bc	1.15 ± 0.10 d
Citronellol	Terpenoids	0.71 ± 0.02 ab	0.32 ± 0.03 d	1.38 ± 0.06 a	0.57 ± 0.15 bc	0.80 ± 0.06 b	0.63 ± 0.08 abc	0.53 ± 0.12 bc	0.45 ± 0.05 cd
Cis-Geraniol	Terpenoids	0.07 ± 0.02 bc	0.11 ± 0.00 a	0.03 ± 0.00 d	0.12 ± 0.02 a	0.08 ± 0.08 b	0.07 ± 0.02 bc	0.04 ± 0.00 cd	0.05 ± 0.00 bcd
Methyl geranate	Terpenoids	nd	0.10 ± 0.08 b	0.31 ± 0.01 a	0.04 ± 0.02 bc	0.11 ± 0.06 b	0.04 ± 0.00 bc	nd	0.07 ± 0.06 bc
5-Methyl-2-furanmethanethiol	Thiols	nd	0.29 ± 0.02 b	nd	0.40 ± 0.08 a	nd	0.12 ± 0.01 c	nd	0.21 ± 0.06 b
Tetradecane	Alkanes	0.23 ± 0.03 a	0.14 ± 0.02 bc	nd	0.04 ± 0.05 de	0.13 ± 0.00 bc	nd	0.18 ± 0.05 ab	0.10 ± 0.01 cd
trans-3,5-dimethyl-1,6-octadiene	Dienes	0.04 ± 0.00 bc	0.06 ± 0.01 bc	0.05 ± 0.01 bc	0.02 ± 0.02 c	0.06 ± 0.01 bc	0.10 ± 0.02 a	0.07 ± 0.01ab	0.06 ± 0.00 b
Humulene epoxide I	Epoxides	0.04 ± 0.00 c	0.06 ± 0.00 bc	0.15 ± 0.02 a	0.10 ± 0.02 b	0.07 ± 0.04 bc	0.05 ± 0.02 c	0.06 ± 0.01 bc	0.05 ± 0.01 c
Octanoic acid	Fatty acids	0.14 ± 0.04 b	0.77 ± 0.53 ab	0.40 ± 0.10 ab	0.90 ± 0.09 ab	0.63 ± 0.06 ab	1.51 ± 1.49 ab	1.21 ± 0.29 ab	2.36 ± 1.65 a

^1^Values are expressed as mean (n = 2) ± standard deviation. The mean values with different letters (a, b, c, d, e, f) within the same row are statistically different (*P* < 0.05). ^2^ nd—not detected.

Beers fermented by *Saccharomyces cerevisiae var. diastaticus* (SAP and SAD) had the highest total volatiles content (TV). The top fermented beers S04P and S04D also were characterised by high TV values, while the the bottom fermented beers S23P and S23D showed the lowest TV. The addition of dark malts in wort production did not have a statistically significant (*P* < 0.05) effect on total volatile content, but was the cause of differences in esters content in beers fermented by *Saccharomyces cerevisiae* (S04), *Saccharomyces cerevisiae var. diastaticus* (SA) and terpenes and terpenoids in beers fermented by S04 and *Saccharomyces pastorianus* (S23) yeast. Dark beers had higher esters content in samples fermented by S04 and SA yeast. The ketones content did not differ between variants. On the contrary, the content of terpenes and terpenoids was significantly higher in pale beers than in dark beers fermented by the S04, S23 and *Saccharomyces cerevisiae* kveik type (KV) strain. The microorganisms used in fermentation strongly differentiate the compositions of volatile compounds in beers. On average, the ale beers S04P and S04D contained 1.5 times higher total volatiles, 1.8 times more aldehydes, and 1.7 times more esters compared to the lager beers S23P and S23D. A difference was also observed when comparing SAP and SAD with the classic bottom fermented beers S23P and S23D. Beers made with *S. cerevisiae var. diastaticus* contained more than 2 times higher TV, with 2.2 times more esters and 2 times more higher alcohols than lager beers.

A key factor in shaping the aromatic profile of beers is the yeast strain used in the alcoholic fermentation process^5^. Volatile compounds that shape the aroma of beer are formed as by-products during the metabolic transformation of the wort components, by which yeast cells are supplied with the compounds necessary for growth and development (amino acids, proteins, nucleic acids, lipids, and others)^11^. The top fermentating yeasts produce a higher total amount of volatile compounds than the bottom fermentating yeasts. Furthermore, ale yeasts can form more esters and higher alcohols than lager yeast, which was also confirmed in our study^9^. Castro et al.^4^ observed that the volatile content in beers increases with increasing fermentation temperature. Each beer fermented by the top fermenting yeast strain, with higher optimal fermentation temperature (S04, KV, and SA), had a higher total amount of identified volatile compounds. Lasanta et al.^14^ confirmed that conducting fermentation at higher temperatures leads to beers with higher concentrations of volatile compounds, including higher alcohols that can be converted to esters.

The content of coloured Maillard reaction compounds affects the sensory properties of beers both directly through the sensory activity of the chemicals generated and by affecting the yeast metabolism products profile. Dack et al.^5^ showed that during the fermentation of dark worts, yeast produces more higher alcohols and fewer esters than during the fermentation of pale worts. They attributed lower ester synthesis to a reduction in enzyme activity or the expression of genes related to ester synthesis^5^. In our research, a significant difference (*P* < 0.05) in total ester content between pale and dark beer made with the same yeast strain was shown for S04, as well as SA. It can be assumed that the effect of malt colour compounds on the biosynthesis of volatiles depended on the yeast strain used in the fermentation.

Tables 2, 3, 4 shows the results of analyses of beers by gas chromatography methods (GC-FID and GC–MS). During the fermentation, higher alcohols and esters are formed concurrently as by-products^15^. Of the identified compounds, the dominant products, regardless of yeast strain and the wort, among the higher alcohols were 3-methylbutanol (11.46–37.52 mg/L), 2-methylpropan-1-ol (6.37–21.84 mg/L) and phenethyl alcohol (4.93–20.15 mg/L). These are the main higher alcohols in the aroma profile of beers fermented by *Saccharomyces* yeast^11,16^. We identified between 13 and 16 higher alcohols in the beers. More higher alcohols were identified in S04D and KVD than in the corresponding pale beers. Differences in higher alcohols content in beers fermented by different yeast strains are confirmed by Dack et al.^5^. The key nutrients for yeast in the wort are carbohydrates and amino acids. The amino acid content is closely related to the sensory characteristics of beers through their participation in the synthesis of higher alcohols by yeast^3^. Moreover, they are involved in Maillard reactions. Due to the use of dark malts, whose production technology includes a more intensive heat treatment, dark worts contain fewer amino acids than pale^5^. It could result in the reduction of higher alcohols in dark beers. However, in our investigation, we did not confirm the effect of using dark speciality malt on total higher alcohols content. Higher alcohols directly affect the sensory qualities of the product and are also precursors to esters synthesis, which are highly flavour active compounds in beer^11^. They are an important part of the beer aroma composition, but they can also negatively affect the quality. Even if individual compounds in the beers analysed are below their sensory thresholds, they can affect the sensory profile of beers through the synergistic effect of volatile components^3^. Both the composition and the proportion of higher alcohols are important in shaping the quality of beer^15^.

Esters are a crucial group of compounds that shape the aroma of beer. They are formed during fermentation, storage and originate from hop cones^17^. Not only brewing raw materials are the source of esters in beer, they are also synthesized during fermentation by yeast^5,17^. At appropriate concentrations, they can impart desirable fruity and floral notes to beer. The synthesis of esters by yeast is related to both transformations during yeast growth and fat metabolism, as well as during fermentation. Among beer esters, we can distinguish a group of acetate esters (ethyl acetate, isoamyl acetate, phenylethyl acetate) and ethyl esters (ethyl hexanoate, ethyl octanoate, and ethyl decanoate). Esters can penetrate the membranes of yeast cells and permeate beer, so an increase in their content is observed during fermentation^11^. Among the esters in the beers we tested, we detected ethyl acetate (6.01–20.07 mg/L), phenethyl acetate (1.81–7.03 mg/L), ethyl octanoate (0.62–1.44 m/L) and isoamyl acetate (0.54–1.05 mg/L) in the highest concentrations. Lasanta et al.^14^ found that the content of phenylethyl acetate in beers fermented at higher temperatures, regardless of the yeast strain tested, was significantly higher compared to beers fermented at lower temperatures. Our results confirmed that phenylethyl acetate was one of the main esters of beer. In addition, we observed that beers that were fermented with S04 (at 18 °C) yeast contained the highest amount of phenylethyl acetate (up to almost 4 times more) among the beers tested. In contrast, high temperature (35 °C) of fermentation by *Saccharomyces cerevisiae* kveik yeast did not increase phenyl acetate content in beers. The volatile compound content of beers is affected by the yeast strain used and the fermentation temperature^14^.

Carbonyl compounds in beer are identified in relatively smaller amounts than the other discussed chemicals. They are formed as products of the Maillard reaction (including Strecker degradation), by lipid oxidation during wort production, and as an intermediate product during the conversion pathway of amino acids to alcohols. One of the most important aldehydes determined in beer is acetaldehyde, which is intermediate in glucose conversion to ethanol during fermentation^11^. In tested beers, furfural (2.60–7.77 mg/L), decanal (1.79–5.61 mg/L), nonanal (1.42–2.79 mg/L) and acetaldehyde diethyl acetal (0.56–2.23 mg/L) were the most abundant aldehyde. Among ketones, 6-methyl-5-hepten-2-one (0.11–0.28 mg/L) and nerylacetone (0.11–0.69 mg/L) were dominant. The furfural content of beer increases with increasing storage time, so it is an important indicator used to analyse the ageing of beer^19,20^. The increase in furfural concentration in beers during ageing depends on the storage conditions and can be up to 50-fold^21^.

Carbonyl compounds are an important indicator of beer ageing. Among the most relevant are 2-furfural (formed by the Maillard reaction during storage) and 2-methylpropanal, 2-methylbutanal , 3-methylbutanal and 2-phenylacetaldehyde, which are products of Stecker degradation or oxidation of higher alcohols^22^. In our beers, of the aforementioned aldehydes, we identified only furfural (2.60–7.77 mg/L). Dark and pale beers fermented by the S04, S23, and KV yeast strain did not differ statistically (*P* < 0.05) in furfural content. Nevertheless, the observed level of this aldehyde was high compared to its content in beers tested by other authors. Čejka et al.^22^ determined that the content of furfural in beers was 5.2–452 µg/L, while Li et al.^20^ observed 0.4–1.91 µg/L of furfural in fresh beer and up to a 470% increase during storage. Higher content of furfural was identified in dark wort than in pale wort. Gibson et al.^23^ found that regardless of the use of dark malts in the production process, the same increase in furfural content was observed during beer ageing^20^.

The acetaldehyde diethyl acetal content in beers was statistically different (*P* < 0.05) for all samples depending on the malt composition. The pale beers S04P and S23P contained more acetaldehyde diethyl acetal than the corresponding dark beers, while KVP and SAP- fewer. The yeast *S. cerevisiae* kveik and *S. cerevisiae var. diastaticus* produced more acetaldehyde diethyl acetal in dark beers than in pale beers. Acetaldehyde is one of the key flavour ingredients in beers, whose effect on beer quality depends on its concentration. It can impart notes of green apple or an unpleasant pungent aroma to beer. Under acidic pH conditions, acetaldehyde reacts with ethanol to form diethyl acetaldehyde, whose aroma is described as fruity. Different brewing yeasts produced different amounts of acetaldehyde diethyl acetal, which was confirmed in our study^24^. Other authors point to the acetaldehyde diethyl acetal concentration in the range of 1.42 to 8.16 mg/L^24^ and 2.72 to 11.63 mg/L^25^. Aldehydes in beer can be formed as a result of lipid and fatty acids oxidation, Maillard reactions, Strecker degradation, oxidation of isohumulones, aldol condensation of short-chain aldehydes, oxidation of higher alcohols, oxidation of long-chain aldehydes, and secretion by yeast^26^. One of the reasons we recorded fewer diethyl acetal acetaldehyde in dark beers may be the antioxidant activity of melanoidins, coloured macromolecular compounds formed at the final stage of the Maillard reaction, which exhibit antioxidant properties^10^. As antioxidants, melanoidins can inhibit the oxidation reaction leading to the formation of acetaldehyde, which can be converted to acetal^26^.

The terpenes and terpenoids in beers are derived from hop. Among hop terpenes and their derivatives in beers, hydrophobic terpene hydrocarbons (β-myrcene, limonene, α-pinene, α-humulene, and β-farnesene) and hydrophilic terpene alcohols (linalool, geraniol, β-citronellol and nerol) are identified^27^. The following compounds classified as terpenes and their derivatives were identified in the beers studied: D-limonene, linalool, citronellol, caryophyllene, (E)-β-farnesene, humulene, and nerolidol. Linalool (1.15–2.05 mg/L), citronellol (0.32–1.38 mg/L) and nerolidol (0.03–0.12 mg/L) were identified in all beers tested. Other terpenes and terpenoids were identified in single samples. The D-limonene content was detected only for samples fermented by yeast S04 and S23. Moreover, pale beers contained more than 8 times the amount of this compound than dark beers. Differences in the content of terpenes and terpenoids between the analysed beers could be attributable to the potential of yeast strains to biotransformation of monoterpenes^27^.

Linalool was considered as an indicator compound in the analysis of the aroma of hops. In addition to linalool, humulene and farnesene were identified in hop oils, as well as the oxidation products of these compounds, citronellol, geraniol, terpineol, which were compounds that shape the hop aroma. Caryophyllene, humulene, and β-farnesene are components of hop products, but their content decreases during the technological processing of wort^28^. As a result of boiling, the content of myrcene and linalool decreases rapidly. The compounds studied that are more resistant to transformation under boiling wort are humulene, humulene epoxide I, β-farnesene, caryophyllene and geraniol^28^. However, hopping is not the only technological step that affects the conversion and loss of terpenoids. These compounds also undergoes biotransformations with the participation of brewer's yeast. The main terpene hydrocarbons in hop essential oils (β- myrcene, α-humulene, and β-caryophyllene) are usually almost completely reduced during the fermentation process by adsorption onto hydrophobic yeast cells and migration into foam during fermentation^29^. This phenomenon explains the absence or low content of hop terpenes and terpenoids in the beers analysed.

**Figure 1:**
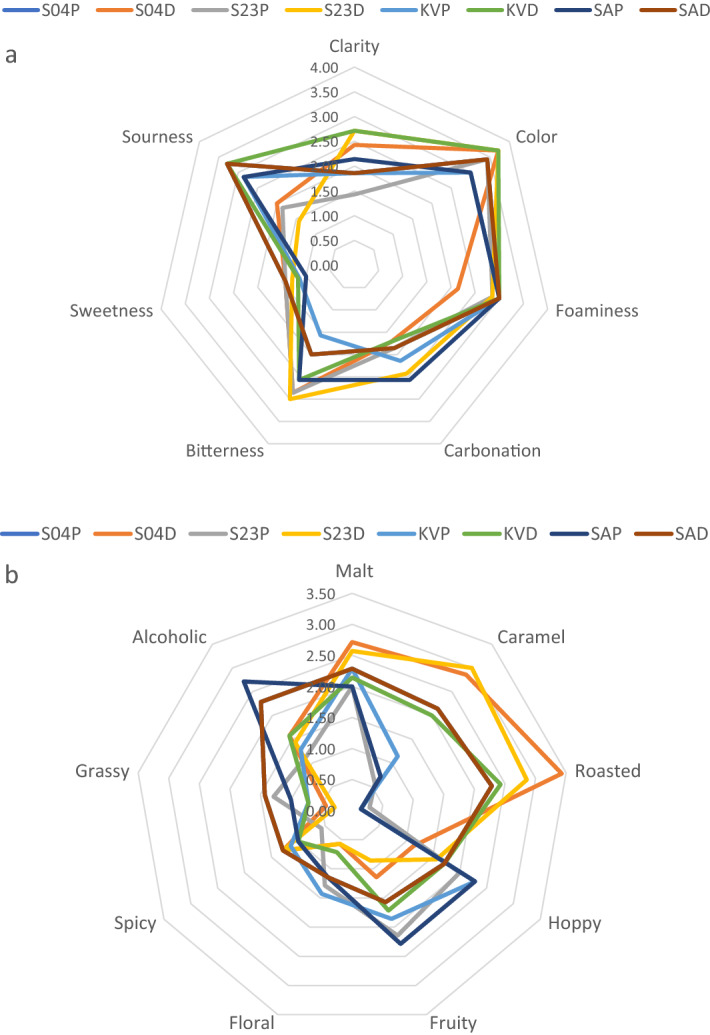
Sensory evaluation of beer, (**a**) Taste (bitter, sweet, sour) and clarity, color, frothiness, carbonation, (**b**) Aroma descriptors.

The beers produced were subjected to sensory analysis. The results were presented in radar graphs (Fig. 1) and added as Supplementary Table S1. All beers were characterised by medium to intense colour, typical of the raw materials used in the production process. The highest foaminess rating had the KVD beer, whose foam was described as good, persistent, fine, and dense. The lowest rating was given to the foaminess of the S04D beer. The beers did not differ (*P* < 0.05) in terms of carbonation. The taste of the beers was analysed for intensity of bitter, sweet and sour taste sensations. The beers analysed were not statistically (*P* < 0.05) different in sweetness. For all beers, it was rated from very weak to moderately perceptible. Dark beers were rated as more bitter than pale beers made with the same yeast strain. Dark specialty malts enhance the bitterness and astringency levels of beers^8^. However, the differences were statistically significant (*P* < 0.05) for each sample. The S23D was rated as the most, while KVP as the least bitter. The most varied ratings in terms of taste among all descriptors used were recorded for sour. The most sour beers were KVD, S04P, SAD, KVP and SAP. Regardless of the malt composition, kveik yeast and *S. cerevisiae var. distaticus* allowed the production of beers with perceptibly higher acidity described as well or even very well perceptible.

The following descriptors were used in the analysis of beer aroma: malt, caramel, roasted, hoppy, fruity, floral, spicy, grassy, and alcoholic. There were no statistically significant differences (*P* < 0.05) between the samples in the perceptibility of malt, hoppy, fruity, floral, spicy, grassy, and alcoholic aromas. However, some trends have been observed. Higher perceptibility of malty, caramel, roasted and spicy aroma was reported in dark beers, while hoppy, fruity, floral and grassy aroma in pale beers. Malt, caramel, and roasted aroma were also rated as well or intensely perceptible in the S04D and S23D beers. Roasted and caramel notes described as undetectable to weakly perceptible characterised pale beers S23P, KVP and SAP. The grassy aroma was very weak to moderately perceptible depending on the variant. S04P, SAP and SAD were identified as the most alcoholic. This is justified because of the highest ethanol content in SAP and SAD among the samples tested^12^.

**Figure 2:**
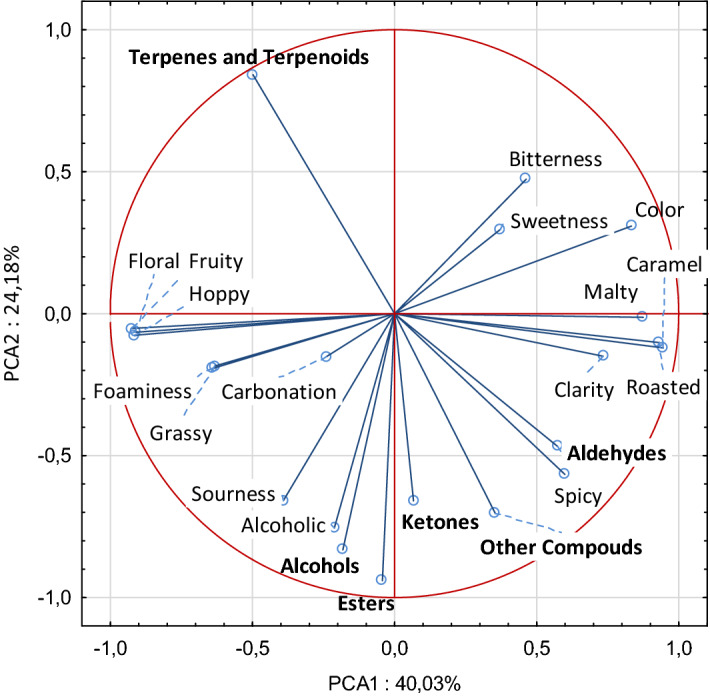
Principal component analysis (PCA) of groups of volatile compounds and beers sensory descriptors.

Principal component analysis (PCA) and correlation analysis (Supplementary Table S2) were performed to better visualize the research results ( Fig. 2). The first two principal components explained 64.21% of the total variation (PC1:40.03 and PC2:24.18%). Correlation analysis between volatile compounds groups showed that higher alcohols were mainly responsible (r = 0.870) for the perception of the alcoholic aroma. There was a significant positive correlation between the content of higher alcohols and esters (r = 0.858). However, there was a negative correlation between terpenes and terpenoids and esters content (r = -0.709) and spice aroma (r = -0.823). Correlations were also observed between the sensory descriptors of the beers. It was shown that the higher bitterness, the lower sensation of sourness (r = -0.724). The caramel aroma was more noticeable with increasing beer colour intensity (r = 0.759) and correlated (r = 0.874) with malty aroma. The sensation of roasted aroma was related to the intensity of caramel aroma (r = 0.956), malty aroma (r = 0.770) and colour intensity (r = 0.860). Roasted, caramel, and malt aromas and colour were strongly linked, forming a group of descriptors shaped mainly by the compositions of malts used in the production of wort. It was also observed that the mentioned group of aroma descriptors along with colour are negatively correlated with the perceptibility of hoppy aromas (hoppy, fruity, floral) (− 0. 962 < r < – 0. 657). A significant correlation was also shown between foaminess and hoppy aroma (r = 0.724) and clarity and floral notes (r = − 0.874).

The composition of the wort and the biochemical transformations that occur during fermentation determine the final quality of the beer. Depending on their concentration, certain flavour compounds can impart positive or negative sensory characteristics. Therefore, it is extremely important to control the fermentation process. The composition of volatile beer compounds depends on the profile of the ingridients of the wort, the conditions of the fermentation process, the yeast strain used, temperature, pressure, oxygen content, and the dose of microorganisms used^9^. The malt aroma is shaped by aldehydes, pyrazines, pyrroles, furans, ketones, acids, and alcohols. The formation of the sweet aroma is attributed to compounds from the aldehyde and furanone group^8^. Pyrazines and pyrroles are responsible for nutty, bread-like, burnt, and roasted aromas. Alcohols and ketones, on the other hand, are involved in shaping the notes perceptible as pungent and sulphurous. Esters and phenols influence the perceptibility of floral, fruity, spicy, and woody notes^8^.

Our research allowed us to characterise the profile of volatile compounds of dark and pale beers fermented by different strains of brewer's yeast. On the basis of the results of chromatographic analyses, we obtained data enabling us to indicate similarities and differences in the distribution of volatile compounds between groups of chemical compounds in the analysed beers. The profile of volatile compounds in beers was found to consist of alcohols, aldehydes, esters, terpenes, terpenoids, ketones, as well as thiols, dienes, fatty acids, and epoxides. Among the beers obtained, the beers fermented by the top fermenting *Saccharomyces cerevisiae var. diastaticus* strain and *Saccharomyces cereviasiae* S04 were characterised by the highest total volatile content, while beers fermented by the bottom-fermenting yeast *Saccharomyces pastorianus* S23 exhibited the lowest. The malt composition did not affect the total content of volatiles, but for some beers it was the cause of significant differences (*P* < 0.05) in the total content of esters, terpenes, and terpenoids. This allows us to conclude that the yeast strain used in the fermentation process influences the profile of volatile compounds of the beers to a greater extent than the malt composition. Differences in the amount of volatile compounds produced between strains are mainly due to variations in esters and alcohols. Beer fermented by *Saccharomyces cerevisiae var. diastaticus* was characterised by an outstandingly high total volatile compound content. Groups of compounds and individual chemicals influence the perceptibility of aroma notes in beers. Therefore, the results obtained in our research will allow a better understanding of formation process of the aroma characteristics of beers.

## Materials and methods

### Biological material

In the experiment, bottom-fermenting brewing yeast *Saccharomyces pastorianus* Saflager S-23 (Fermentis, Lasaffre, France)^30^, and top-fermenting *Saccharomyces cerevisiae* Safale S-04 (Fermentis, Lasaffre, France)^31^, *Saccharomyces cerevisiae* Voss kveik (Lallemand, Canada)^32^ and *Saccharomyces cerevisiae var. diastaticus* Belle Saison (Lallemand, Canada)^33^ were used.

*S. pastorianus* Saflager S-23, *S. cerevisiae* Safale S-04, and *S. cerevisiae* Voss were characterised by high flocculation and sedimentation ability in contrast to *S. cerevisiae var. diastaticus* Belle Saison. The yeast strains used varied in their attenuation capacity. *S. cerevisiae var. diastaticus* Belle Saison was characterised by a remarkably high ability to attenuate wort sugars 84–94%, due to its potential to ferment dextrins^33^. The attenuation level for the remaining yeast strains used was lower, ranging from 74–84%^30–32^. Moreover, a distinguishing feature of *S. cerevisiae var. diastaticus* Belle Saison is also its ability to decarboxylate hydroxycinnamic acids, thus being classified as a POF + (phenolic off flavor) yeast^33^.

### Raw materials

The following raw materials were used in the technological process of beer production: pilsner barley malt (Viking Malt, Strzegom, Poland), dark chocolate barley malt (Viking Malt, Strzegom, Poland), Marynka hop (Polish Hops, Karczmiska Pierwsze, Poland) and Lubelski hop (Polish Hops, Karczmiska Pierwsze, Poland).

### Brewing Technology

The technological process of beer production was carried out according to Paszkot & Kawa-Rygielska^12^ considering the following technological stages: infusion mashing, gravity filtration of the mash, batch sparge with water, boiling of the wort with hops, cooling, filtration, division into 2 L samples, inoculation with brewer's yeast and fermentation. In the pale wort production process, 100% pilsner barley malt (Viking Malt, Strzegom, Poland) was used. Dark wort was produced with 90% pilsner barley malt and 10% chocolate dark barley malt (Viking Malt, Strzegom, Poland). The pale wort contained 10.78 ± 0.13%w/w of extract and had a colour of 8.4 ± 0.36 EBC, while the dark wort contained 11.45 ± 0.18%w/w of extract and had a colour of 85.7 ± 1.7 EBC.The worts tested were not statistically significantl (*P* < 0.05) different in the sum of identified carbohydrates (maltose, maltotriose, glucose and dextrins)^12^. The colour of the worts was analysed using by spectrophotometric method by measuring absorbance at 430 nm (DMA 4500 M, Anton Paar, Graz, Austria). The extract content of the worts was measured using a Densito 30PX densitometer (Metler Toledo, Columbus, USA). Worts were boiled with Marynka hop (7,9% α-acids, dose 1 g/L, 60 min; Polish Hops, Karczmiska, Poland) and Lubelski hop (4% α-acids, dose 1 g/L, 10 min; Polish Hops, Karczmiska, Poland).The worts (2 L) were inoculated with one of four selected strains of brewer yeast at a dose according to the manufacturer's recommendations (1 g of dried biomass per litre of wort). Fermentation was carried out at the temperature optimal for the yeast strain, in accordance with the manufacturer's recommendations and industry practice for 7 days at 18 °C for S04P and S04D samples, 12 °C for S23P and S23D samples, 35 °C for KVP and KVD samples, and 18 °C for SAD and SAP samples. After the separation of the yeast sediment, secondary fermentation was carried out for another 7 days. The beers were then bottled in 0.5 L bottles. During bottling, glucose was added at a dose of 2 g/L to allow refermentation. The maturation of the beer was carried out at 4 °C for 28 days. Basic physicochemical parameters of beers have previously been described^12^. The beers were characterised by the following ethanol content: S04P—4.51%v/v, S04D—4.71%v/v, S23P—4.73%v/v, S23D—4.66%v/v, KVP—4.80%v/v, KVD—4.58%v/v, SAP—5.60%v/v i SAD—5.79%v/v. The real extract of the beers was as follows: S04P—3.75%w/w , S04D—4.38%w/w, S23P—3.70%w/w, S23D—4.56%w/w, KVP—3.34%w/w, KVD—4.20%w/w, SAP—2.23%w/w, SAD –2.58%w/w^12^. During the technological process, microbiological control was carried out in the following stages: inoculation, fermentation, maturation. No microbial contamination was found.

### Analysis of volatile compounds by gas chromatography

#### Gas chromatography with flame ionising detection (GC-FID)

The volatile compounds of the beers were analysed using gas chromatography coupled with flame ionisation detection (GC-FID) technique, using a GC2010 Plus apparatus with FID-2010 detector equipped with a headspace autosampler (HS-20) (Shimadzu Corporation, Kyoto, Japan) and a CP-WAX 57 CB column (50 m × 0.32 mm ID × 0.2 µm) (Agilent Technologies, Santa Clara, CA, USA). Before analyses, beers were degassed by shaking on an orbital shaker, mixed with diatomaceous earth (1 g/100 mL of beer) and filtered through a paper filter. Beer samples (10 mL) were transferred to glass headspace vials (20 mL). The vials were then conditioned and shaken in a headspace autosampler oven (40 °C, 20 min.) before 1 mL of the sample was transferred to the HS-20 headspace loop connected to the column, with the following parameters: holding time − 0.5 min, equilibration time − 0.1 min, loading time − 0.5 min, equilibration time − 0.1 min, injection time − 0.5 min, and the total GC analysis time − 60 min. The following temperature program was used for gas chromatography analysis: 40 °C (3 min), increase (5 °C/min) to 80 °C, maintain 80 °C (3 min), increase (10 °C/min) to 140 °C, maintain 140 °C (9 min), increase (20 °C/min) to 160 °C and maintain 160 °C (4 min). The total cycle time was 34 min. An initial pressure of 100 kPa was used, the initial flow 6.6 mL/min, the initial column flow 0.33 mL/min, the initial linear velocity 11.8 cm/s, and the purge flow was set at 3 mL/min. Helium was used as the carrier gas. The FID was operated at 280 °C with a sampling rate of 40 ms and a hold time of 34 min. H_2_ flow into the FID was at a rate of 50 mL/min, the air flow was 400 mL/min, and the helium flow was 30 mL/min. Data were integrated and quantified in LabSolutions software (Shimadzu Corporation, Kyoto, Japan). The identification of compounds was performed using analytical standards. Quantification was performed using external standards based on a standard curve with five calibration points (the coefficient of determination R^2^ was greater than or equal to 0.999).

#### Gas chromatography mass spectrometry (GC–MS)

The volatile compounds were adsorbed on solid phase microextraction fibre (SPME) according to Gasiński et al. ^34^ using 30 µL of internal standard (IS) (1 mg of 2-undecanone per 1 dm^3^ of cyclohexane) and 2 cm^3^ of beer. The extraction of volatiles was performed for 20 min at a temperature of 40 °C. Volatile compounds were analysed by gas chromatography mass spectrometry using a GC-2010 Plus chromatograph coupled with a GCMS-QP2010 SE mass spectrometer (Shimadzu, Kyoto, Japan), equipped with a ZB-5 column (Phenomenex, Torrance, CA, USA) (30 m length × 0.25 mm inner diameter × 0.25 μm layer thickness). Helium was used as the carrier gas (1.78 cm^3^/min, initial pressure 100 kPa). The injection port was maintained at 195 °C. The volatiles were desorbed from the fibre (1 cm long DVB/CAR/PDMS fibre with 50/30 µm thickness of stationary phase; Supelco, Bellefonte, PA, USA) in the injection port for 2 min. The following oven temperature programme for the GC analysis was used: 40 °C for 1 min, ramp up (8 °C/min) to 195 °C; hold (5 min). The ion source temperature was 250 °C and the interface temperature was 195 °C. Scanning was performed in the range of 35–350 m/z using 70 mV electron ionisation, with an event time of 0.3 s (scan rate of 1111). Mass spectral analysis was used to identify volatile compounds. Identification was carried out by comparative analysis of retention indices with Kovats standards and NIST17 chemical standard libraries. Peak integration was performed using the Shimadzu PostRun Analysis software (Shimadzu, Kyoto, Japan).

### Sensory analysis

Sensory analysis of the beers was carried out using the author's questionnaire. The following characteristics of the beers were evaluated: clarity, colour, foaminess, and carbonation. Beers were evaluated for the perceptibility of bitter, sweet, and sour flavours. The aroma of the beers was evaluated for the detectability of malty, caramel, roasted, hoppy, fruity, floral, spice, grassy and alcoholic notes. The sensory panel was attended by 7 people, including two men and five women. All participants in the study were qualified and experienced in specialised sensory analysis of beers. A table showing the scores of the descriptors assessed in the sensory analysis is attached as Supplementary Table S3. Participation in sensory evaluation of products was voluntary. Each participant was informed of and agreed to the principles of the analysis. The data is confidential and will not be used without their knowledge.

### Statistics

Parameters were compared with the one-way analysis of variance (ANOVA) at α = 0.05 using Statistica 13.5 (StatSoft, Tulsa, OK, USA). Duncan's test (*P* < 0.05) was used to detect statistically significant differences between mean scores. The tables show the mean and standard deviation values. Principal component analysis (PCA) was also performed to compare the results of the gas chromatography analysis and the sensory characteristics of beer.

## Supplementary Information

Supplementary Information 1.

Supplementary Information 2.

Supplementary Information 3.

## Data Availability

The data presented in this study are available on request from the corresponding author.
