# Supplementary material for: Evaluation of volatile compound profiles and sensory properties of dark and pale beers fermented by different strains of brewing yeast
Source: Sci Rep. 2023 Apr 25;13:6725. doi: 10.1038/s41598-023-33246-4 (PMC10130024; doi:10.1038/s41598-023-33246-4)
Supplement: Supplementary file 3 — Supplementary Information 3. [file 41598_2023_33246_MOESM3_ESM.pdf]

Supplementary Information 3 Sensory evaluation of beers - quality descriptors and explanation of the scoring used

**Feature**	**Score**				
**Scale**	**0**	**1**	**2**	**3**	**4**
Clarity	Very cloudy	Cloudy	Slightly cloudy	Clear	Very clear
Color	Undesirable, deviating from the natural color of the raw materials	Slightly deviating from the natural color of the raw materials	Neutral, characteristic of the raw materials used	Moderately intense, characteristic of the raw materials used	Intensive, characteristic of the raw materials used
Foaminess	No foam	Insufficient, unstable, low, large bubbles	Sufficient, not very persistent, not very abundant, medium bubble	Good, persistent, fine bubble, abundant, dense	Very good, persistent, fine bubble, very abundant, dense
Saturation	No saturation	Insufficient, very weak, barely perceptible, short-lived	Sufficient, not very intense, short-lived	Good, moderately intense, long-lived	Very good, intense, long-lived
Flavor	Not perceptible	Very weakly perceptible	Medium perceptible	Well perceptible	Intensely perceptible
Aroma	Not perceptible	Very weakly perceptible	Medium perceptible	Well perceptible	Intensely perceptible
